# Presidential address: introduction of smart device-based testing and item exposure policy for Korea Health Personnel Licensing Examination

**DOI:** 10.3352/jeehp.2017.14.5

**Published:** 2017-03-26

**Authors:** Chang Hwi Kim

**Affiliations:** Korea Health Personnel Licensing Examination Institute; Hallym University, Korea

The Korea Health Personnel Licensing Examination Institute has done its best to establish a more stable and examinee-centered system after its re-launch as a government-supported special foundation on December 23, 2015. As one of the results of this process, an item development center for the national health personnel licensing examinations will be opened this year, which has been one of the most coveted projects of the institute. This center will be located in Chungju, Chungchongbuk-do, and will provide a more comfortable and secure place for the professionals who prepare examination items (Fig. 1). It will be able to accommodate 120 individuals, and to be used as an intensive training center for them. I hope that this center will be a locus of activities to improve the credibility of the national licensing examinations.

In 2017, a smart device-based test (SBT), which has been in preparation since 2006, will be administered for the first time. This year, there will be a SBT in the licensing examination for emergency medical technicians. In 2006, a preliminary study was conducted on the implementation of a computer-based test (CBT), and suggestions were made for a CBT to be implemented in professions with a small number of examinees, for a CBT center to be constructed, and for an item bank database for the CBT to be developed. In 2007, a seminar on CBT for the staff members of the institute was held, and a task force team was suggested. In 2008, a team tasked with developing a CBT was launched. This team suggested the development of a computerized adaptive test (CAT) instead of a CBT, because CATs are suitable for tailored tests and make cheating impossible. In 2009, the CAT-first policy was abandoned due to the difficulty of developing an item bank and the test equating problem. It was suggested that CAT could be reconsidered after the development of a CBT. In 2010, an action plan for CBT development was developed, including the following 3 items: first, a mock test shall be made available in any CBT center in Korea for professions with a small number of examinees; second, an information system for item and exam management shall be developed, as well as a supervision system; and third, staff members shall visit other institutes in Korea and abroad where CBT has been implemented.

In 2011, an administrative unit responsible for this was launched under the Bureau of Test Management. At a consulting meeting on CBT in April 2011, Prof. Younghwan Kim from the Department of Education at Pusan National University suggested ubiquitous-based testing (UBT) instead of a desktop-based CBT. UBT belongs to the general category of CBT, but is accessible through smart devices, such as smartphones or tablets. It was decided to introduce UBT instead of CBT because UBT has the advantages of providing a comfortable test environment, allowing effective test management, and being cost-saving. In July 2011, a budget of 92,847,000 Korean won was permitted to be used for the development of a mock SBT, which was renamed from UBT due to its stress on the use of smart-device. In August 2011, a memorandum of understanding between this institute and NSDevil Co. was signed to establish mutual cooperation in the implementation of SBT in health personnel licensing examinations in Korea. A CBT promotion committee was constituted in August 2011, with 11 members. The members were elected with a term extending to the first implementation of SBT in any profession. In November 2011, the first mock licensing examination was conducted for 400 medical technology students. In October 2012, CBT was conducted for 200 medical technology students, as well as SBT for 200 medical technology students. In November 2012, CBT for 200 dental students was conducted. Although the mock tests were first conducted for medical technology and dentistry, those professions refused to accept SBT or CBT at that time. Therefore, priority was given to SBT for emergency medical technicians and physicians. For the emergency medical technician licensing examination, it was decided to implement SBT starting in late 2017. Mock tests would be conducted from 2014 to 2016. For the medical licensing examination, SBT was decided to be implemented in early 2020. Mock tests would be conducted from 2015 to 2019. In this year, SBT will be an offline test, with devices not connected to the internet. Therefore, examinees’ responses will be gathered after the examination. The absence of an internet/intranet connection was implemented due to the security problems that may occur with a wireless connection. A shortcoming of the offline test is that CAT cannot be implemented, because the examinees’ ability cannot be estimated in real time when examinees answer questions. This is the first implementation of SBT for high-stakes testing in the world. I hope it is executed without any technical problems.

Another important policy is making the content of the test items of all 24 licensing examinations available to the public starting in 2020. This was requested by the members of the National Assembly of Korea during the inspection period of this institute. Up to this point, only items from the medical licensing examination have been available since 2012. Although it is not desirable to make the test items available, due to concerns about maintaining the item bank and the preparation of CAT, the institute should attempt this policy. It will evaluate the effects of this policy after its implementation.

I hope the research results originating from the institute’s policy changes, as well as the examination data, can be presented to the world through the *Journal of Educational Evaluation for Health Professions*. This will assist in the development of high-stakes examinations for the medical health professions. Finally, I would like to express my gratitude to the editorial committee members and hope that everyone visiting the *Journal of Educational Evaluation for Health Professions* will be full of health and happiness.

## Figures and Tables

**Fig. 1.**
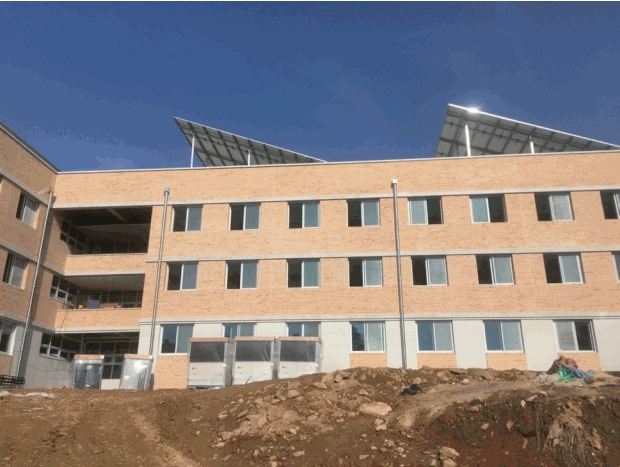
The item development center for the national health personnel licensing examinations in Chungju, Chungchongbuk-do.

